# Oblique Lumbar Interbody Fusion Combined With Anterolateral Fixation and Cement Augmentation for the Treatment of Degenerative Lumbar Diseases in the Elderly Population: A Retrospective Study

**DOI:** 10.1111/os.14315

**Published:** 2024-12-03

**Authors:** Weiqi Han, Lei He, Fei Wang, Xiaofeng Zhao, Cong Jin

**Affiliations:** ^1^ Department of Orthopedics Shaoxing People's Hospital Shaoxing China

**Keywords:** bone cement, intervertebral disc disease, spinal fusion, spinal stenosis

## Abstract

**Objectives:**

Cage subsidence is a common complication of oblique lumbar interbody fusion (OLIF), particularly in elderly patients with osteoporosis or osteopenia. While bilateral pedicle screw fixation (BPS) is effective in reducing subsidence, it is associated with longer operative times, increased blood loss, and greater tissue trauma. In contrast, anterolateral fixation (AF) is less invasive but linked to higher subsidence rates. Ensuring both minimal invasiveness and adequate stability in OLIF‐assisted fixation remains a significant challenge. This study aimed to evaluate the efficacy of combining AF with cement augmentation (AF + CA) in reducing cage subsidence and improving clinical outcomes compared with AF and BPS.

**Methods:**

A retrospective analysis was conducted on 138 elderly patients with degenerative lumbar diseases treated with OLIF. Patients were divided into three groups: AF + CA (32 patients), AF (32 patients), and BPS (74 patients). Clinical and radiographic outcomes were compared among the groups, and logistic regression analyses were performed to identify risk factors for cage subsidence after OLIF.

**Results:**

At 1 year postoperatively, the disc height of the AF + CA group was significantly greater than that of the AF group. The cage subsidence rate in the AF + CA group was 24.3%, significantly lower than that in the AF group (48.8%, *p* < 0.05) and comparable to the BPS group (30.4%). Survivorship curve analysis showed better outcomes in reducing cage subsidence in the AF + CA group compared with the AF group, with no significant difference between the AF + CA and BPS groups. Compared with the AF + CA and BPS groups, the AF group had significantly higher grades and severity of cage subsidence. Fusion rates at 1 year were 91.9% in the AF + CA group, 90.2% in the AF group, and 95.1% in the BPS group, with no significant differences. The AF + CA group had significantly shorter operative times, less intraoperative blood loss, lower VAS scores at 3 days and 1 year postoperatively, and lower ODI scores at 3 days and 3 months compared with the BPS group. Multivariate regression analysis revealed that AF was a significant risk factor for cage subsidence, with an odds ratio of 3.399 compared with AF + CA.

**Conclusions:**

AF + CA effectively reduces cage subsidence in OLIF surgeries, offering results comparable to BPS while providing advantages such as shorter surgical time, reduced blood loss, and improved early postoperative outcomes. AF + CA is a viable alternative, especially for elderly patients with comorbidities who may not tolerate the longer operative durations or greater blood loss associated with BPS.

## Graphical abstract

Cement augmentation significantly enhances screw stability, strengthens endplates, and reduces the risk of cage subsidence in oblique lumbar interbody fusion combined with AF, particularly benefiting elderly patients with degenerative lumbar conditions.

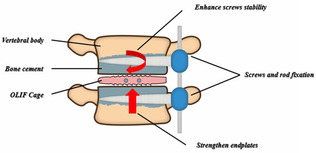

## 1 Introduction

Oblique lumbar interbody fusion (OLIF) is recognized as a minimally invasive lumbar fusion technique that offers advantages such as minimal surgical trauma, rapid postoperative recovery, and reduced neural interference [1, 2]. However, cage subsidence frequently occurs postoperatively, leading to a decrease in disc height, compromised indirect decompression, the recurrence of foraminal and spinal stenosis, and potential revision surgery [3]. Combining OLIF with bilateral pedicle screw fixation (BPS) has been shown to significantly increase postoperative lumbar stability, effectively supporting the cage and reducing the risk of subsidence [4]. Biomechanical studies by Cai et al. indicated that OLIF with BPS maintains lumbar stability, prevents cage subsidence, and preserves disc height better than OLIF with unilateral pedicle screw or anterolateral fixation (AF) [5]. Despite its effectiveness, BPS requires intraoperative repositioning, additional posterior incisions, longer surgical times, and increased blood loss [6, 7]. Additionally, posterior screw placement can damage the paraspinal muscles and soft tissues, increasing postoperative back pain and delaying rehabilitation [6, 7].

Research conducted by Wang et al. has demonstrated that OLIF combined with AF is a relatively safe and effective surgical approach for managing degenerative lumbar diseases [2]. Liu and Feng reported that anterior lateral screw–rod fixation minimizes the operative time, blood loss, radiation exposure, and soft tissue damage, achieving single‐stage interbody fusion and fixation through a small incision [8]. However, cage subsidence remains a primary complication, particularly in elderly and osteoporotic patients. Wang et al. reported that increasing osteoporosis significantly increases the maximum stress on the endplates of the fusion segment, thereby increasing the potential risk of cage subsidence [9]. Additionally, Kotheeranurak et al. reported that age > 60 years and bone mineral density (BMD) < −2.5 are risk factors for cage subsidence post‐OLIF [10]. Therefore, strategies to reduce the risk of cage subsidence following OLIF with AF in elderly individuals are crucial.

Several methods have been reported to address this issue effectively, providing valuable insights into surgical optimization. For example, Wang et al. demonstrated that the trajectory of anterolateral pedicle screws, including their angle and position, is closely correlated with cage subsidence. It has been shown that inserting screws parallel to each other and as close to the endplate as possible while ensuring that the cage remains within the range of the superior and inferior screws constitutes the optimal implantation strategy for AF [2]. Additionally, Li et al. investigated the management of lumbar spinal stenosis with osteoporosis via OLIF combined with AF and stress endplate augmentation. The findings revealed that the cage subsidence rate in the stress endplate augmentation group was 13.3%, which was significantly lower than the 56.67% rate observed in the AF alone group [7].

The use of cement‐augmented pedicle screws in transforaminal lumbar interbody fusion (TLIF) for treating osteoporotic lumbar degenerative diseases has been well documented. Song et al. reported that cement‐augmented pedicle screws are more effective than conventional pedicle screws in enhancing fusion rates, maintaining disc height, reducing screw loosening, and improving long‐term outcomes [11]. Similarly, Wang et al. suggested that cement‐augmented pedicle screws have greater fusion rates and lower rates of screw loosening than conventional screws [12]. However, no literature exists on the application of cement‐augmented screws in OLIF combined with AF for treating lumbar degenerative diseases in elderly individuals.

The purposes of the study were designed as follows: (i) To evaluate the efficacy of AF with cement augmentation (AF + CA) in reducing the incidence of cage subsidence following OLIF, in comparison to AF and BPS. (ii) To assess the potential of AF + CA to enhance clinical outcomes, including operative time, intraoperative blood loss, postoperative pain, and functional recovery, compared with BPS and AF. (iii) To identify the risk factors for cage subsidence in elderly patients undergoing OLIF, with a focus on the influence of AF + CA.

## 2 Methods

### 2.1 Study Design

This study retrospectively analyzed 138 elderly patients with degenerative lumbar diseases treated with OLIF at Shaoxing People's Hospital from January 2017 to December 2021. According to the methods used for auxiliary internal fixation, the patients were divided into the AF + CA group, the AF group, and the BPS group. The clinical and radiographic outcomes of the three groups were compared. Univariate and multivariate regression analyses were performed to examine the associations between cage subsidence and various independent factors. This study was approved by the Ethics Committee of Shaoxing University Affiliated First Hospital (No. 202405201), and informed consent was obtained from all patients.

### 2.2 Participants

The inclusion criteria for this study were patients over 60 years of age; those who underwent OLIF with auxiliary internal fixation for degenerative lumbar diseases; individuals with preoperative diagnoses of lumbar disc herniation, spinal stenosis, or spondylolisthesis; those with a preoperative BMD of −1.0 or lower; and those with a follow‐up period exceeding 1 year. Patients were excluded if they had a history of previous lumbar surgeries, severe spinal stenosis or grade III or IV spondylolisthesis necessitating posterior decompression, involvement of more than three surgical segments, severe hepatic or renal dysfunction, primary or secondary spinal tumors, or secondary osteoporosis resulting from long‐term glucocorticoid use.

### 2.3 Surgery and Postoperative Care

All surgeries were performed by two surgical teams. After induction of general anesthesia, the patient was placed in the right lateral decubitus position with the left hip flexed to reduce tension on the psoas muscle. Following routine disinfection and draping, a 5 cm oblique incision was made 5 cm anterior to the fluoroscopically determined center of the intervertebral disc. The skin and subcutaneous tissue were incised, followed by blunt dissection through the external oblique, internal oblique, and transversus abdominis muscles to reach the peritoneum. The peritoneum, along with the extraperitoneal fat, was retracted ventrally to expose the anterior edge of the psoas muscle. At the disc level, the psoas muscle was dissected posteriorly to expose the intervertebral disc. After confirming the surgical segment under fluoroscopy, a working channel was established. Through the channel, the disc was excised, the cartilage endplates were prepared, and the bone graft bed was readied. An appropriately sized OLIF cage filled with allograft bone was inserted. The center point of the cage was identified, and the screw insertion points were marked 0.5 cm from both the upper and lower endplates. The dissection range along both sides of the upper and lower endplates should not exceed 1 cm, particularly for the lower endplate, to avoid damaging the segmental vessels. The direction of screw insertion should be parallel to the endplate and the cage, and as close to the endplate as possible to enhance screw strength. The screws should be as long as possible, and positioned ~0.5 cm from the opposite cortex, but without penetrating it, to reduce the risk of cement leakage and prevent improper screw placement that could injure the contralateral segmental vessels. After screw insertion was completed, rods were placed in patients in the AF group and locked directly. Patients in the AF + CA group had 1.5 mL of bone cement injected into each of the upper and lower vertebrae through hollow screws via a pusher under C‐arm guidance, followed by rod placement and locking. Patients in the BPS group were positioned prone, and BPS was performed via the Wiltse approach.

After the wound drainage tube was removed 48 h postoperatively, the patients were permitted to ambulate with the support of a soft brace and participate in lower limb and back muscle strengthening exercises. Basic osteoporosis treatment consisting of 600 mg of Caltrate D once daily and 0.25 μg of calcitriol once daily was administered to all patients.

### 2.4 Baseline Data

Baseline data, including patient age, gender, body mass index (BMI), BMD, diagnosis, surgical segment, number of surgical segments, smoking and alcohol history, and hypertension and diabetes history, were obtained directly from the electronic medical records system.

### 2.5 Radiographic Evaluation

Radiographic assessments included anterior disc height (AH), posterior disc height (PH), disc height (DH), coronal disc angle (CDA), sagittal disc angle (SDA), foraminal height (FH), and lumbar lordosis (LL) [10, 13, 14]. AH was defined as the distance between the anterior inferior edge of the upper vertebral body and the anterior superior edge of the lower vertebral body on lateral X‐ray images. PH was defined as the distance between the posterior inferior edge of the upper vertebral body and the posterior superior edge of the lower vertebral body on lateral X‐ray images. DH was defined as the average of the AH and PH. CDA was defined as the angle between the inferior endplate of the upper vertebrae and the superior endplate of the lower vertebrae on anteroposterior X‐ray images. SDA was defined as the angle between the inferior endplate of the upper vertebrae and the superior endplate of the lower vertebrae on lateral X‐ray images. FH was defined as the distance between the highest point of the inferior edge of the pedicle of the upper vertebra and the lowest point of the superior edge of the pedicle of the lower vertebra on lateral X‐ray images. LL was defined as the angle between the vertical line to the superior endplate of L1 and the vertical line to the superior endplate of S1 on lateral X‐ray images. Radiographic assessments were performed preoperatively, 3 days postoperatively, and 1 year postoperatively. All the radiographic measurements were performed by two radiologists, and the results were averaged. To assess inter‐rater reliability, intraclass correlation coefficients (ICCs) were calculated for each measurement. The ICC values for AH, PH, CDA, SDA, FH, and LL were found to be 0.952, 0.850, 0.877, 0.908, 0.873, and 0.940, respectively, indicating excellent to good reliability for all measurements.

The morphology of the endplate, which included both flat and concave types, was assessed on preoperative sagittal T2‐weighted MR images. Additionally, the evaluation of endplate lesions, including Schmorl's nodes and Modic changes, was performed on preoperative sagittal T2‐weighted MR images. The degree of paraspinal muscle degeneration was assessed on preoperative axial T2‐weighted MR images at the midline of L3. According to previous studies [15], the grading system was applied as follows: Grade 0 signifies a normal condition; Grade 1 indicates that the fatty infiltration area occupies 0%–10% of the paraspinal muscle area; Grade 2 denotes an infiltration area of 10%–50%; and Grade 3 denotes an infiltration area of > 50% of the paraspinal muscle area.

### 2.6 Operative Outcomes

Operative data, including operative time and intraoperative blood loss, were obtained directly from the surgical records in the electronic medical records system.

### 2.7 Cage‐Related Outcomes

The cage‐related data included the cage height, cage length, and cage location. Cage height and cage length were obtained directly from the surgical records in the electronic medical records system. Cage location was defined as the ratio of the distance from the center point of the cage to the anterior edge of the intervertebral space to the distance from the center point of the cage to the posterior edge of the intervertebral space, which was measured on postoperative lateral X‐rays. These measurements were performed by two radiologists, and the average value was obtained. The ICC value for cage location was 0.937, indicating excellent to good reliability.

### 2.8 Clinical Outcomes

Clinical outcomes were evaluated using the back visual analog scale (VAS) score, leg VAS score, and Oswestry Disability Index (ODI) score. The assessments were conducted preoperatively, 3 days postoperatively, 3 months postoperatively, and at 1‐year postoperative intervals. The VAS [16], a simple tool for measuring pain intensity, consists of a line labeled “no pain” to “worst pain imaginable,” where patients mark their pain level. This mark is then converted to a numerical value, typically ranging from 0 to 10. The ODI [17], a questionnaire designed to measure a patient's permanent functional disability, focuses on the impact of lower back pain on daily activities.

### 2.9 Cage Subsidence

The definition of cage subsidence is established through the assessment of postoperative X‐ray lateral views, where the presence of the cage sinking into either the superior or inferior endplate is observed [18]. In accordance with prior literature, cage subsidence grading was also performed [19, 20]. The rate of DH loss was calculated using the following formula: (postoperative DH—DH at 1 year postoperative)/postoperative DH * 100%. The classification criteria are as follows: Grade 0 denotes a DH loss rate of 0%–24%, Grade 1 represents a loss rate of 25%–49%, Grade 2 signifies a loss rate of 50%–74%, and Grade 3 denotes a loss rate of 75%–100%. Grades 0 and 1 are categorized as mild cage subsidence, whereas Grades 2 and 3 are categorized as severe cage subsidence. Evaluations for cage subsidence were conducted at 1 month, 3 months, 6 months, and 1 year postoperatively.

### 2.10 Interbody Fusion

All patients underwent an assessment of interbody fusion status at 1 year postoperatively. The evaluation was conducted by one senior orthopedic surgeon and one radiologist. Interbody fusion was defined as present if both evaluators concurrently determined fusion. According to previous studies [21, 22, 23], interbody fusion requires the following criteria: (1) Reconstructed CT sagittal images revealed evident trabecular bone formation, with bone bridges extending from the cage to the upper and lower endplates and no radiolucent zones between the cage and endplates. (2) Continuous trabecular bone formation surrounding the cage was observed, bridging the upper and lower endplates. (3) Flexion and extension X‐rays demonstrated a range of motion at the fused segment of < 5°.

### 2.11 Statistical Analysis

Statistical analysis was performed using SPSS (version 19.0; SPSS Inc., Chicago, IL) on the Windows platform. The chi‐square test was used to compare gender, number of surgical segments, history of diabetes and hypertension, and cage subsidence rates among the three groups. Yates' correction was applied to assess surgical segment, diagnosis, history of smoking and alcohol consumption, grade of cage subsidence, severity of cage subsidence, fusion rate, and cage length among the three groups. Cage location was evaluated using one‐way ANOVA, with a normal distribution confirmed by the Shapiro–Wilk normality test and variance homogeneity assessed by Levene's test. Age, BMI, BMD, operative time, intraoperative blood loss, and cage height were compared between groups using the Kruskal–Wallis test. Two‐way repeated‐measures ANOVA was employed to compare the VAS score, ODI, AH, PH, DH, CDA, SDA, FH, and LL among the three groups. To assess the inter‐rater reliability of radiographic measurements, ICCs were calculated for each parameter. ICCs were calculated using a two‐way random‐effects model with absolute agreement. Logistic regression was utilized to model the relationships between cage subsidence and the independent variables. Survival curve analysis of cage subsidence among the three groups was conducted using both Cox regression and log‐rank tests. The significance level was set at 0.05.

## 3 Results

### 3.1 Baseline Data

A total of 138 patients (180 surgical segments) were included in this retrospective study, with 32 patients (37 surgical segments) in the AF + CA group, 32 patients (41 surgical segments) in the AF group, and 74 patients (102 surgical segments) in the BPS group. There were no significant differences in the baseline data, including age, gender, BMI, BMD, diagnosis, surgical segment, number of surgical segments, history of hypertension and diabetes, smoking status, or alcohol consumption, among the three groups (Table 1).

**TABLE 1:** Comparison of baseline data among the three groups.

Characteristics	AF + CA group	AF group	BPS group	*p*	Statistical value
*N*	32	32	74		
Age, median (IQR)	69.5 (64.75, 74.25)	69 (66, 71)	70 (67, 74)	0.299	2.417
Gender, *n* (%)				0.265	2.656
Male	11 (8.0%)	14 (10.1%)	38 (27.5%)		
Female	21 (15.2%)	18 (13.0%)	36 (26.1%)		
BMI, median (IQR)	23.52 (21.98, 24.22)	24.63 (22.35, 25.53)	23.49 (21.66, 25.29)	0.407	1.797
BMD, median (IQR)	−1.40 (−2.53, −1.18)	−1.60 (−2.45, −1.10)	−1.30 (−2.38, −1.10)	0.905	0.199
Diagnosis, *n* (%)				0.862	1.297
Spinal stenosis	20 (14.5%)	17 (12.3%)	45 (32.6%)		
Lumbar disc herniation	4 (2.9%)	5 (3.6%)	7 (5.1%)		
Spondylolisthesis	8 (5.8%)	10 (7.2%)	22 (15.9%)		
Surgical segment, *n* (%)				0.942	0.775
L23	1 (0.6%)	1 (0.6%)	5 (2.8%)		
L34	10 (5.6%)	12 (6.7%)	30 (16.7%)		
L45	26 (14.4%)	28 (15.6%)	67 (37.2%)		
Number of surgical segments, *n* (%)				0.070	5.311
1	27 (19.6%)	23 (16.7%)	46 (33.3%)		
2	5 (3.6%)	9 (6.5%)	28 (20.3%)		
Diabetes, *n* (%)				0.806	0.431
Yes	4 (2.9%)	5 (3.6%)	13 (9.4%)		
No	28 (20.3%)	27 (19.6%)	61 (44.2%)		
Hypertension, *n* (%)				0.699	0.716
Yes	16 (11.6%)	14 (10.1%)	39 (28.3%)		
No	16 (11.6%)	18 (13%)	35 (25.4%)		
Smoking, *n* (%)				0.379	1.940
Yes	3 (2.2%)	3 (2.2%)	13 (9.4%)		
No	29 (21.0%)	29 (21.0%)	61 (44.2%)		
Alcohol, *n* (%)				0.140	3.935
Yes	1 (0.7%)	3 (2.2%)	12 (8.7%)		
No	31 (22.5%)	29 (21%)	62 (44.9%)		

Abbreviations: AF + CA: anterolateral fixation and cement augmentation; AF: anterolateral fixation; BMD: bone mineral density; BMI: body mass index; BPS: bilateral pedicle screw fixation; IQR: Interquartile range.

### 3.2 Radiographic Outcomes

No significant differences in AH, PH, or DH were observed among the three groups at the preoperative or postoperative time points. Additionally, no significant differences in AH, PH, or DH were found between the AF + CA and BPS groups at 1 year postoperative. No significant differences were observed among the three groups regarding CDA, SDA, FH, or LL at the preoperative, postoperative, or 1‐year postoperative assessments (*p* > 0.05).

One year postoperatively, AH was significantly greater in the AF + CA group (14.76 ± 3.03) than in the AF group (12.68 ± 3.62) (*p* < 0.05). Similarly, the AH in the BPS group (15.42 ± 3.17) was significantly greater than that in the AF group (*p* < 0.05). The PH in the AF + CA group (9.26 ± 1.93) was significantly greater than that in the AF group (7.81 ± 2.29) (*p* < 0.05); likewise, the PH in the BPS group (9.58 ± 1.96) was significantly greater than that in the AF group (*p* < 0.05). The DH in the AF + CA group (12.00 ± 2.26) was significantly greater than that in the AF group (10.24 ± 2.64) (*p* < 0.05); similarly, the DH in the BPS group (12.50 ± 2.23) was significantly greater than that in the AF group (*p* < 0.05) (Table 2).

**TABLE 2:** Comparison of radiographic data among the three groups.

Characteristics	AF + CA group	AF group	BPS group
AH			
Preop, mean ± SD	12.58 ± 2.49	11.30 ± 2.53	12.38 ± 2.93
Postop, mean ± SD	16.06 ± 2.96	16.10 ± 2.32	16.52 ± 2.86
At 1 year, mean ± SD	14.76 ± 3.03^a^	12.68 ± 3.62^b^	15.42 ± 3.17
PH			
Preop, median (IQR)	6.80 (6.30, 8.50)	7.10 (6.10, 7.60)	7.00 (5.80, 8.60)
Postop, mean ± SD	10.20 ± 2.23	10.30 ± 1.67	10.21 ± 2.04
At 1 year, mean ± SD	9.26 ± 1.93^a^	7.81 ± 2.29^b^	9.58 ± 1.96
DH			
Preop, mean ± SD	9.91 ± 1.79	9.05 ± 1.48	9.77 ± 2.24
Postop, median (IQR)	13.30 (12.45, 14.30)	13.35 (12.45, 14.05)	13.48 (11.81, 14.86)
At 1 year, mean ± SD	12.00 ± 2.26^a^	10.24 ± 2.64^b^	12.50 ± 2.23
CDA			
Preop, median (IQR)	1.20 (0.50, 3.10)	1.10 (0.50, 2.20)	1.10 (0.50, 2.43)
Postop, median (IQR)	0.60 (0.40, 1.20)	0.90 (0.30, 1.60)	0.80 (0.30, 1.38)
At 1 year, median (IQR)	1.00 (0.50, 1.30)	1.10 (0.70, 2.00)	1.00 (0.60, 1.50)
SDA			
Preop, median (IQR)	6.20 (4.70, 9.10)	5.50 (3.40, 7.70)	6.95 (4.53, 9.80)
Postop, median (IQR)	6.60 (5.10, 9.50)	8.00 (5.90, 9.90)	8.05 (5.43, 9.90)
At 1 year, mean ± SD	7.11 ± 3.02	7.10 ± 2.73	7.66 ± 3.15
FH			
Preop, median (IQR)	18.20 (16.90, 19.50)	17.30 (16.40, 19.40)	17.95 (16.53, 19.28)
Postop, median (IQR)	22.40 (20.60, 24.10)	22.00 (19.90, 23.90)	21.70 (19.30, 23.60)
At 1 year, median (IQR)	20.70 (19.00, 22.80)	19.40 (17.80, 20.80)	21.00 (18.80, 22.45)
LL			
Preop, mean ± SD	45.99 ± 9.90	42.37 ± 9.47	43.69 ± 14.42
Postop, mean ± SD	44.82 ± 8.17	41.64 ± 10.04	40.77 ± 11.97
At 1 year, mean ± SD	44.96 ± 7.28	41.41 ± 8.61	41.98 ± 10.29

Abbreviations: AF + CA: anterolateral fixation and cement augmentation; AF: anterolateral fixation; AH: anterior height of disc space; BPS: bilateral pedicle screw fixation; CDA: coronal disc angle; DH: disc height; FH: foraminal height; IQR: interquartile range; LL: lumbar lordosis; PH: posterior height of disc space; SD: standard deviation; SDA: sagittal disc angle.

^a^ *p* < 0.05, AF + CA group compared with the AF group.

^b^ *p* < 0.05, AF group compared with the BPS group.

### 3.3 Operative Outcomes

The operative time in the AF + CA group was 88 (81, 97.25) min, which was significantly lower than that in the BPS group (195.5 (169.25, 226.75) min) (*p* < 0.05). Similarly, the operative time in the AF group was 89.5 (82.75, 102.5) min, which was also significantly lower than that in the BPS group. However, no significant difference in operative time was observed between the AF + CA group and the AF group.

The intraoperative blood loss in the AF + CA group was 42.5 (30, 50) mL, which was significantly less than that in the BPS group (50 (50, 100) ml) (*p* < 0.05). However, there were no significant differences in intraoperative blood loss between the AF + CA group and the AF group or between the AF group and the BPS group (*p* > 0.05) (Table 3).

**TABLE 3:** Comparison of operative and cage‐related data among the three groups.

Characteristics	AF + CA group	AF group	BPS group
Operative time(min), median (IQR)	88 (81, 97.25)^a^	89.5 (82.75, 102.5)^b^	195.5 (169.25, 226.75)
Intraoperative blood loss (mL), median (IQR)	42.5 (30, 50)^a^	50 (50, 65)	50 (50, 100)
Cage height, median (IQR)	12 (12, 13)	12 (12, 12.25)	12 (12, 13)
Cage length, *n* (%)			
45	17 (12.3%)	18 (13.0%)	40 (29.0%)
50	12 (8.7%)	14 (10.1%)	32 (23.2%)
55	3 (2.2%)	0 (0.0%)	2 (1.4%)
Cage location, mean ± SD	1.08 ± 0.25	1.16 ± 0.31	1.10 ± 0.29

Abbreviations: AF + CA: anterolateral fixation and cement augmentation; AF: anterolateral fixation; BPS: bilateral pedicle screw fixation; IQR: interquartile range; SD: standard deviation.

^a^ *p* < 0.05, AF + CA group compared with the BPS group.

^b^ *p* < 0.05, AF group compared with the BPS group.

### 3.4 Cage‐Related Outcomes

There were no statistically significant differences (*p* > 0.05) observed among the three groups in terms of cage‐related outcomes, including cage height, cage length, or cage location (Table 3).

### 3.5 Clinical Outcomes

There were no statistically significant differences in the VAS score among the three groups at baseline or 3 months postoperatively. However, at 3 days postoperatively, the back VAS scores were significantly lower in both the AF + CA and AF groups than in the BPS group (*p* < 0.05). At 1 year postoperatively, the back VAS score in the AF + CA group was also significantly lower than that in the BPS group (*p* < 0.05).

Similarly, there were no statistically significant differences in leg VAS scores among the three groups at baseline or at 3 months postoperatively. However, at 3 days postoperatively, the leg VAS score in the AF + CA group was significantly lower than that in the BPS group (*p* < 0.05). At 1 year postoperatively, the leg VAS scores in the AF + CA group were significantly lower than those in both the AF and BPS groups (*p* < 0.05).

**FIGURE 1:**
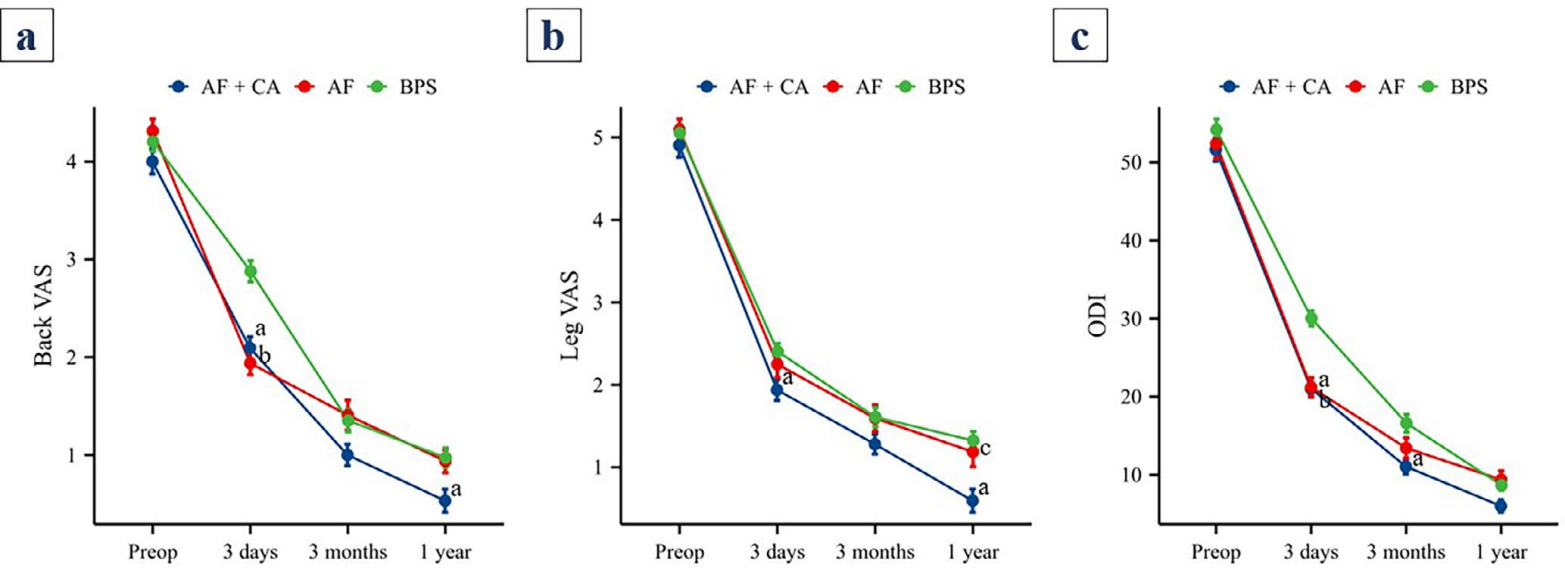
Comparison of clinical outcomes among the three groups. (a) *p* < 0.05, AF + CA group compared with the BPS group. (b) *p* < 0.05, AF group compared with the BPS group; (c) *p* < 0.05, AF group compared with the AF + CA group. AF + CA: anterolateral fixation and cement augmentation; AF: anterolateral fixation; BPS: bilateral pedicle screw fixation.

Furthermore, there were no statistically significant differences in the ODI among the three groups at baseline or 1 year postoperatively. However, at 3 days postoperatively, the ODI was significantly lower in both the AF + CA and AF groups than in the BPS group (*p* < 0.05). At 3 months postoperatively, the ODI in the AF + CA group was also significantly lower than that in the BPS group (*p* < 0.05) (Figure 1).

### 3.6 Cage Subsidence Rates and Fusion Rates

**FIGURE 2:**
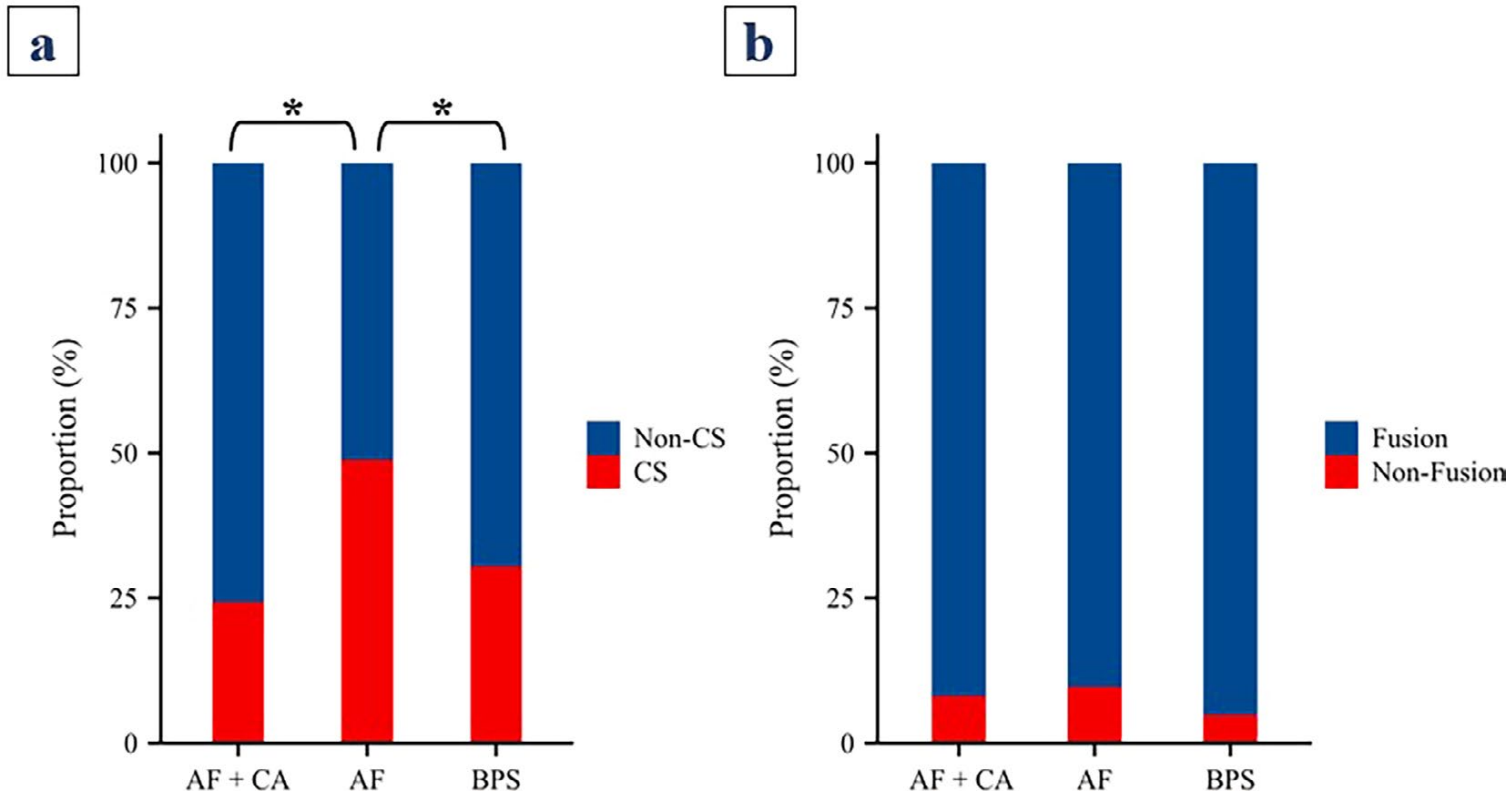
Comparison of cage subsidence rates and fusion rates among the three groups. *p < 0.05, compared with the AF group. AF + CA: anterolateral fixation and cement augmentation; AF: anterolateral fixation; BPS: bilateral pedicle screw fixation; CS: cage subsidence; non‐CS: non‐cage subsidence.

The cage subsidence rate was 24.3% (9/37) in the AF + CA group, which was significantly lower than the 48.8% (20/41) in the AF group (*p* < 0.05). The BPS group presented a cage subsidence rate of 30.4% (31/102), which was also significantly lower than that of the AF group (*p* < 0.05). However, there was no significant difference in cage subsidence rates between the AF + CA group and the BPS group (*p* > 0.05) (Figure 2a).

At 1 year postoperatively, the fusion rate was 91.9% (34/37) in the AF + CA group, 90.2% (37/41) in the AF group, and 95.1% (97/102) in the BPS group. There were no statistically significant differences in the fusion rates among the three groups (*p* > 0.05) (Figure 2b).

### 3.7 Survival Curve Analysis of Cage Subsidence

**FIGURE 3:**
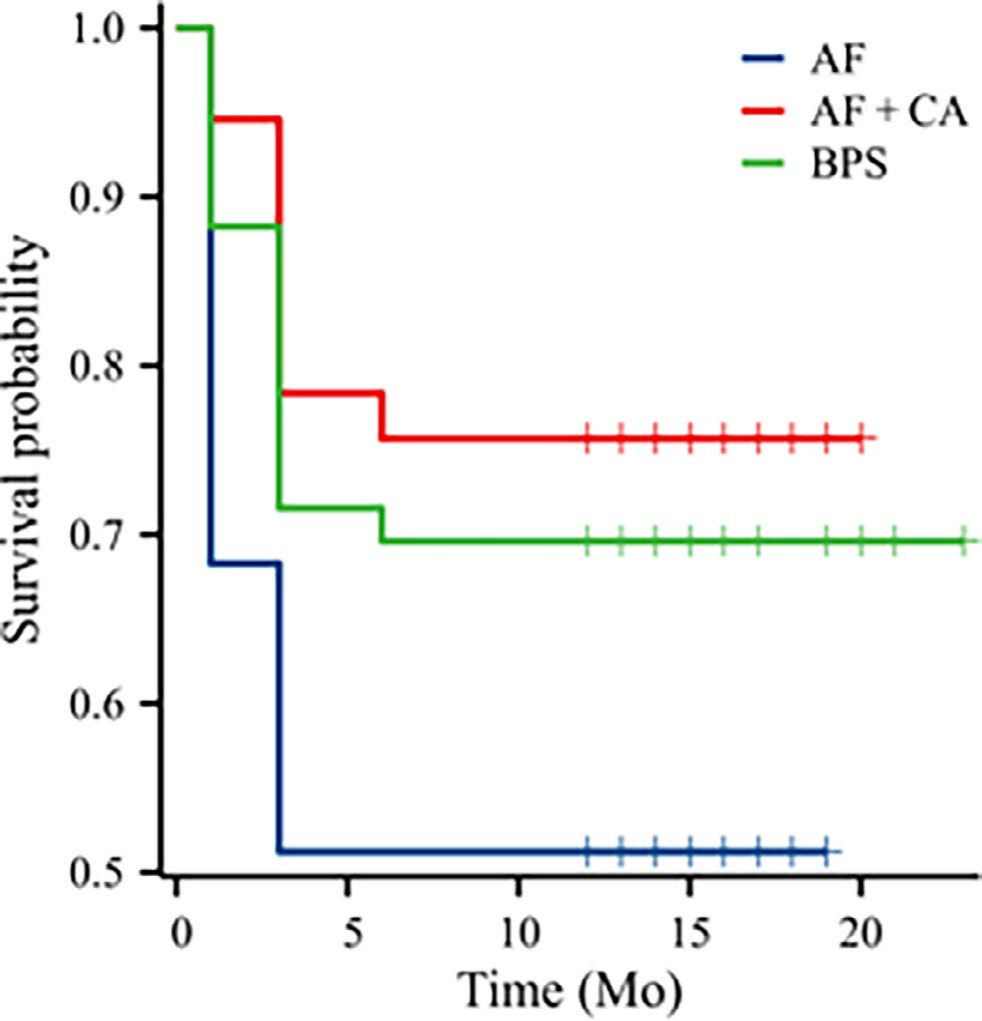
Survival curve analysis of cage subsidence among the three groups. AF + CA: anterolateral fixation and cement augmentation; AF: anterolateral fixation; BPS: bilateral pedicle screw fixation; Mo: months.

Survival curve analysis of cage subsidence among the three groups was conducted using both Cox regression and log‐rank tests. The AF + CA group demonstrated better outcomes in terms of reducing cage subsidence than did the AF group. Cox regression analysis revealed that, with the AF group as the reference group, the AF + CA group demonstrated a significantly lower risk of cage subsidence (HR 0.38, 95% CI 0.173–0.837, *p* = 0.016) than the AF group. Similarly, compared with the AF group, the BPS group also had a lower risk of cage subsidence (HR 0.50, 95% CI 0.288–0.886; *p* = 0.017). The log‐rank test further supported these findings, showing that the AF + CA group had a significantly lower hazard ratio than the AF group (HR 0.42, 95% CI 0.205–0.878; *p* = 0.015). However, no statistically significant differences were found between the AF + CA group and the BPS group or between the AF group and the BPS group (Figure 3).

### 3.8 Severity of Cage Subsidence

The grading and severity of cage subsidence in both the AF + CA and BPS groups were significantly different from those in the AF group (*p* < 0.05), whereas there was no significant difference between the AF + CA and BPS groups (Table 4).

**TABLE 4:** Comparison of the severity of cage subsidence among the three groups.

Characteristics	AF + CA group	AF group	BPS group	*p*	Statistical value
*N*	9	20	31		
Grade, *n* (%)				< 0.001	44.903
0	8 (13.3%)	1 (1.7%)	29 (48.3%)		
1	1 (1.7%)	12 (20%)	2 (3.3%)		
2	0 (0%)	7 (11.7%)	0 (0%)		
3	0 (0%)	0 (0%)	0 (0%)		
Severity, *n* (%)				< 0.001	15.849
Mild	9 (15%)	13 (21.7%)	31 (51.7%)		
Severe	0 (0%)	7 (11.7%)	0 (0%)		

Abbreviations: AF + CA: anterolateral fixation and cement augmentation; AF: anterolateral fixation; BPS: bilateral pedicle screw fixation.

### 3.9 Univariate Analysis of Risk Factors for Cage Subsidence

According to the univariate analysis, female gender (OR = 2.368, 95% CI 1.155–4.854, *p* = 0.019), BMD (OR = 0.515, 95% CI 0.335–0.791, *p* = 0.002), no history of hypertension (OR = 2.412, 95% CI 1.188–4.900, *p* = 0.015), concave endplate morphology (OR = 2.385, 95% CI 1.243–4.575, *p* = 0.009), and Modic change (OR = 0.245, 95% CI 0.081–0.740, *p* = 0.013) were identified as risk factors for cage subsidence following OLIF surgery (Table 5).

**TABLE 5:** Univariate analysis of risk factors for cage subsidence.

Characteristics	Univariate analysis	
	Odds ratio (95% CI)	*p*
Fixation		
AF + CA	Reference	
AF	2.556 (0.907–7.204)	0.076
BPS	1.468 (0.594–3.627)	0.405
Fusion level		
L23	1.771 (0.377–8.317)	0.469
L34	1.599 (0.812–3.149)	0.174
L45	Reference	
Age	0.981 (0.927–1.039)	0.520
Gender		
Male	Reference	
Female	2.368 (1.155–4.854)	0.019
BMI	1.094 (0.959–1.249)	0.181
BMD	0.515 (0.335–0.791)	0.002
Diagnosis		
Spinal stenosis	Reference	
Lumbar disc herniation	0.310 (0.082–1.171)	0.084
Spondylolisthesis	0.723 (0.330–1.583)	0.417
Number of fusion levels		
1	Reference	
2	2.097 (1.000–4.398)	0.050
Diabetes		
Yes	2.280 (0.906–5.735)	0.080
No	Reference	
Hypertension		
Yes	Reference	
No	2.412 (1.188–4.900)	0.015
Smoking		
Yes	Reference	
No	1.365 (0.485–3.845)	0.556
Alcohol		
Yes	0.991 (0.338–2.908)	0.987
No	Reference	
Height of cage	0.992 (0.739–1.333)	0.960
Length of cage		
45	3.246 (0.364–28.900)	0.291
50	1.897 (0.210–17.158)	0.569
55	Reference	
Location of cage	1.205 (0.418–3.473)	0.730
Endplate morphology		
Flat	Reference	
Concave	2.385 (1.243–4.575)	0.009
Endplate lesion		
No	Reference	
Modic change	0.245 (0.081–0.740)	0.013
Schmorl's node	0.945 (0.264–3.385)	0.931
Paravertebral muscle degeneration		
Grade 0	Reference	
Grade 1	0.332 (0.087–1.263)	0.106
Grade 2	1.200 (0.325–4.427)	0.784
Grade 3	1.200 (0.246–5.844)	0.821
Pre AH	0.976 (0.872–1.091)	0.664
Pre PH	0.992 (0.846–1.163)	0.920
Pre CDA	1.058 (0.949–1.179)	0.311
Pre SDA	0.963 (0.890–1.041)	0.341
Pre FH	1.094 (0.965–1.239)	0.160
Pre LL	0.979 (0.951–1.007)	0.135

Abbreviations: AF + CA: anterolateral fixation and cement augmentation; AF: anterolateral fixation; AH: anterior height of the disc space; BMD: bone mineral density; BMI: body mass index; BPS: bilateral pedicle screw fixation; CDA: coronal disc angle; DH: disc height; FH: foraminal height; LL: lumbar lordosis; PH: posterior height of the disc space; SDA: sagittal disc angle.

### 3.10 Multivariate Analysis of Risk Factors for Cage Subsidence

**FIGURE 4:**
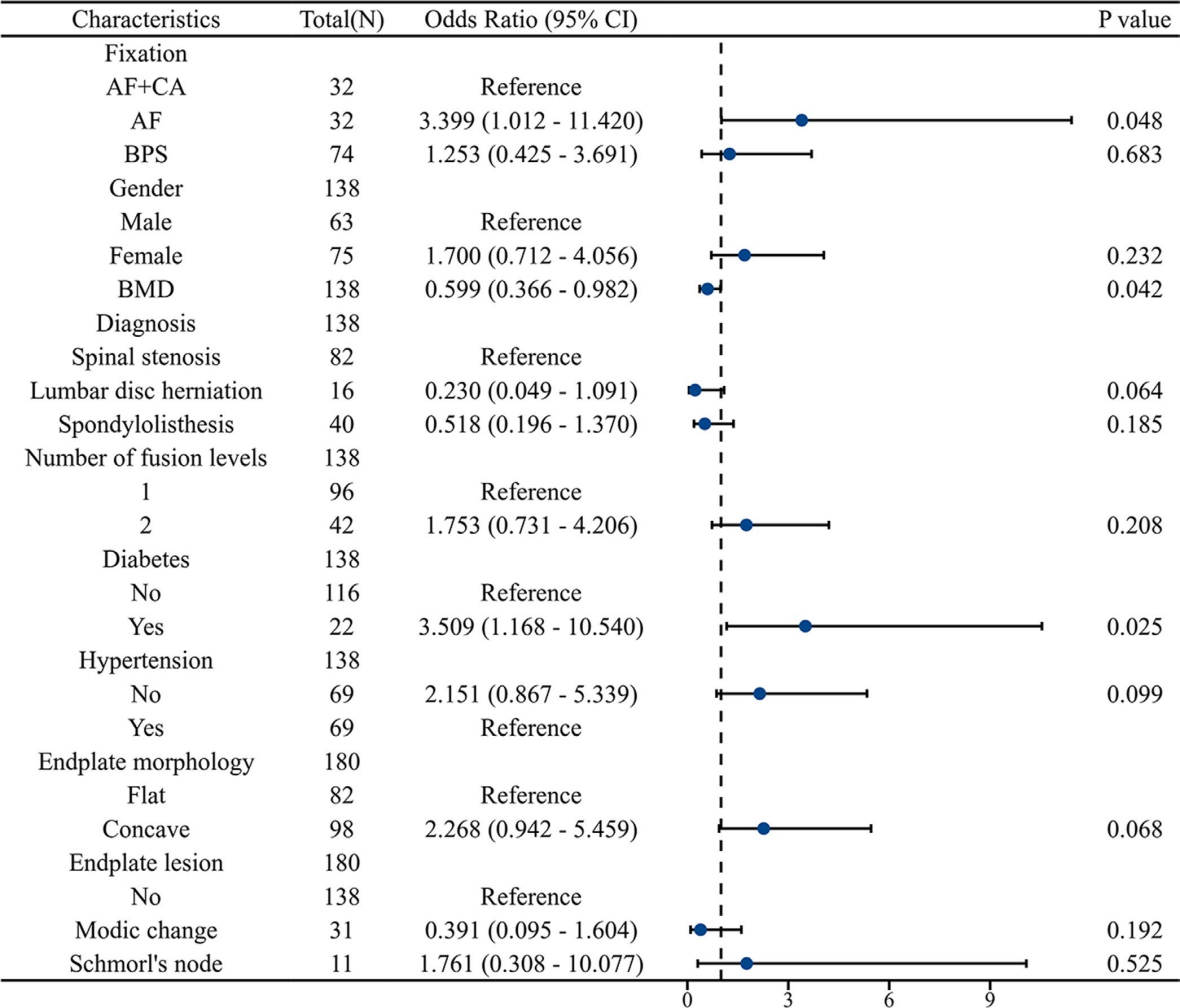
Multivariate analysis of risk factors for cage subsidence. AF + CA: anterolateral fixation and cement augmentation; AF: anterolateral fixation; BMD: bone mineral density; BPS: bilateral pedicle screw fixation.

**FIGURE 5:**
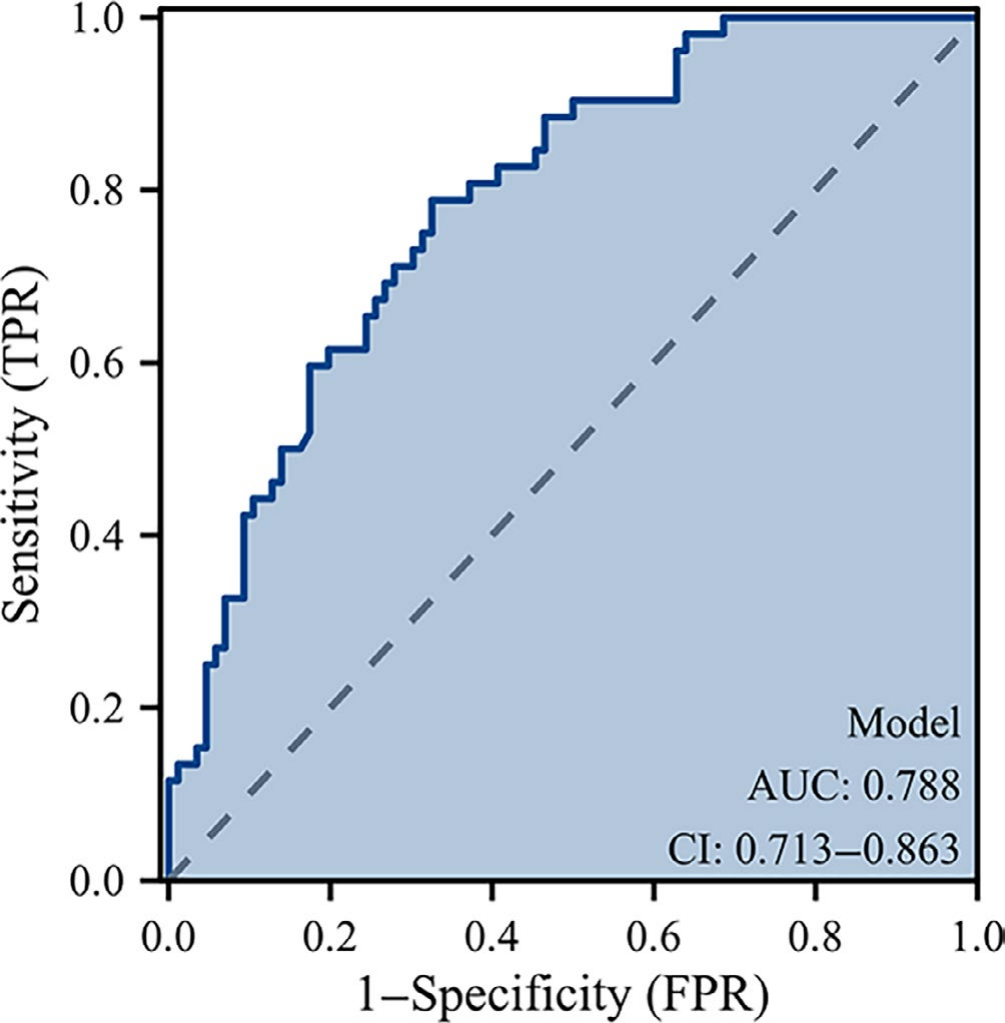
Receiver operating curve. Receiver operating characteristic curve demonstrating the accuracy of the model for predicting cage subsidence. The area under the curve (AUC) was 0.788 (95% CI 0.713–0.863).

**FIGURE 6:**
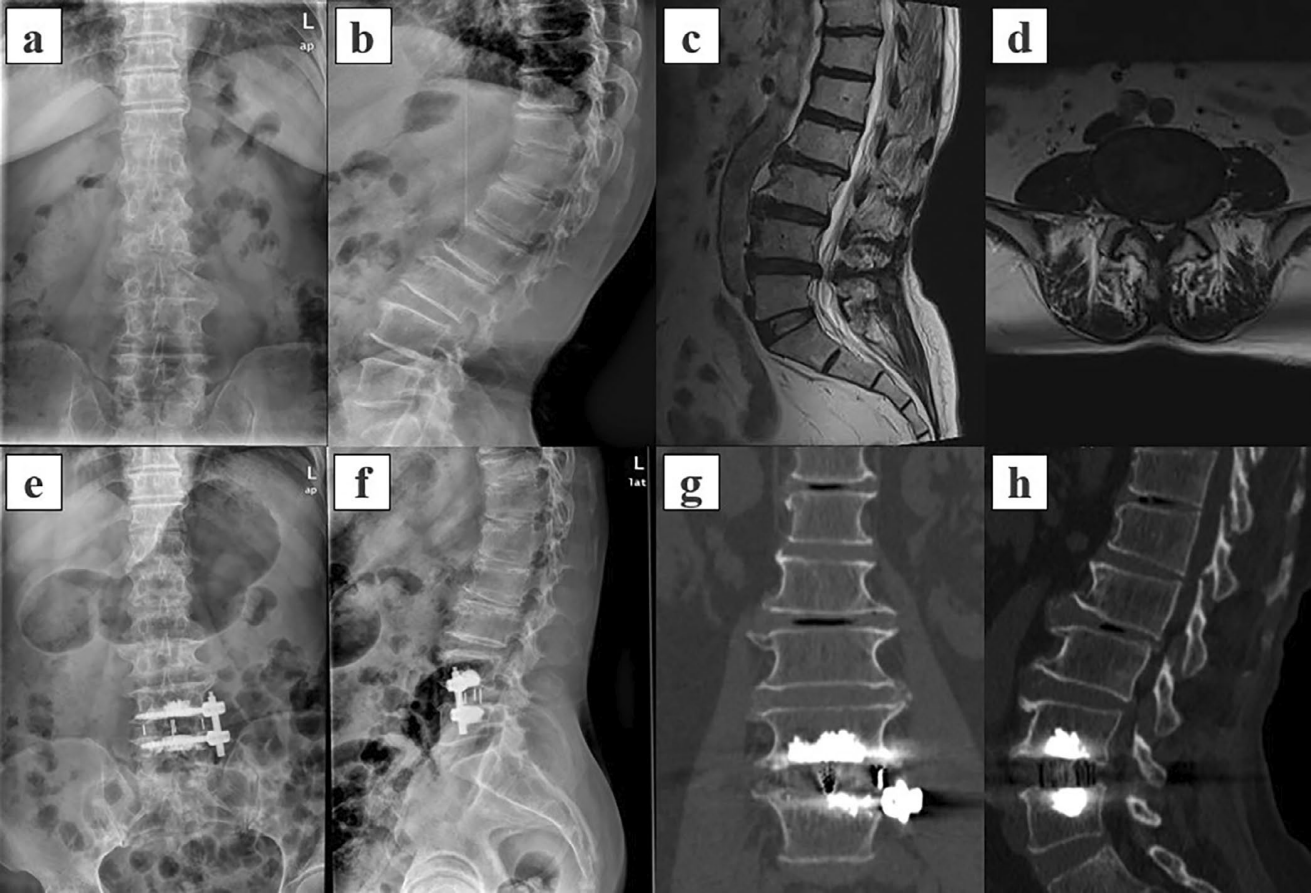
Typical case from the AF + CA group. A 68‐year‐old female patient was preoperatively diagnosed with L45 spinal stenosis and underwent L45 OLIF with AF + CA. She was followed up for 14 months postoperatively. (a, b) Preoperative X‐ray images show a preoperative AH of 15.6 mm, pH of 9.3 mm, CDA of 4.0°, SDA of 7.1°, FH of 18.2 mm, and LL of 41.1°. (c, d) Preoperative MR image indicating spinal stenosis at L4/5. (e, f) Postoperative X‐ray images show a postoperative AH of 15.9 mm, pH of 10.3 mm, CDA of 0.2°, SDA of 10.1°, FH of 24.4 mm, and LL of 28.3°. Postoperative X‐rays indicated excellent positioning of the cage and anterolateral screws, with satisfactory cement dispersion. (g, h) At the last follow‐up, 14 months postoperatively, 3D reconstructed CT indicated successful interbody fusion with no significant cage subsidence. AF + CA: anterolateral fixation and cement augmentation; AH: anterior height of disc space; CDA: coronal disc angle; FH: foraminal height; LL: lumbar lordosis; PH: posterior height of disc space; SDA: sagittal disc angle.

In the multivariate analysis, AF fixation (OR = 3.399, 95% CI 1.012–11.420, *p* = 0.048), BMD (OR = 0.599, 95% CI 0.366–0.982, *p* = 0.042), and diabetes (OR = 3.509, 95% CI 1.168–10.540, *p* = 0.025) were identified as risk factors for cage subsidence following OLIF surgery (Figure 4). The area under the curve (AUC) was 0.788 (95% CI 0.713–0.863) (Figures 5 and 6).

## 4 Discussion

The main findings of this study indicate that AF + CA is an effective method for preventing cage subsidence in OLIF surgeries. Comparable benefits to BPS were observed, with the additional advantages of shorter operative times, reduced intraoperative blood loss, and improved early postoperative pain relief and functional outcomes. While BPS is traditionally regarded as the optimal choice for minimizing cage subsidence in OLIF, the results of this study demonstrate that AF + CA can achieve comparable outcomes, with a more favorable recovery profile, particularly in the elderly population.

### 4.1 Cement Augmentation in Reducing the Rate of Cage Subsidence

A key finding of this study was that cement augmentation significantly reduced the incidence of cage subsidence following anterolateral screw fixation. Previous literature has reported cage subsidence rates in the AF group ranging from 52.6% to 56.67% [7, 24, 25].. In this study, the rate of subsidence in the AF group was 48.8%, aligning with these earlier findings. Notably, the subsidence rate in the AF + CA group was only 24.3%, significantly lower than that of the AF group in both this study and prior reports. A more detailed analysis of cage subsidence was performed in this study, comparing subsidence rates between the AF + CA and AF groups. Cox regression and log‐rank test results demonstrated a significantly lower risk of cage subsidence in the AF + CA group. Additionally, the AF group exhibited significantly higher grades and greater severity of subsidence compared with the AF + CA group. Multivariate regression analysis identified AF as a substantial risk factor for cage subsidence, with an odds ratio of 3.399, using AF + CA as the reference. These results underscore that cement augmentation greatly enhanced intervertebral stability, reinforced endplate strength, and effectively reduced the risk of cage subsidence.

Additionally, AF + CA achieved results comparable to BPS in reducing the rate of cage subsidence. BPS is considered the optimal method for enhancing OLIF stability [6, 7]. Biomechanical analyses by Cai et al. and Xu et al. demonstrated that BPS is superior to AF in maintaining lumbar stability, resisting cage subsidence, and preserving disc height. BPS has been shown to more effectively reduce intervertebral stress and spinal ligament tension, thereby better maintaining segmental stability and providing an ideal environment for fusion [5, 26]. Zhang et al. reported that the subsidence rate in the BPS group was significantly lower than that in the AF group, which had a subsidence rate of 52.6% [25]. In the present study, the subsidence rate in the AF + CA group was 24.3%, which was comparable to that of the BPS group. Moreover, the Cox regression and log‐rank test results indicated that there was no significant difference in the risk of cage subsidence between the AF + CA and BPS groups. Additionally, there were no statistically significant differences in subsidence grade or severity between the AF + CA and BPS groups. These findings further confirm that cement augmentation significantly improves intervertebral stability, increases endplate strength, reduces the risk of cage subsidence, and achieves results similar to those of BPS.

### 4.2 AF + CA Minimizes Surgical Trauma and Improves Postoperative Recovery

AF + CA not only achieves stability comparable to BPS but also minimizes surgical trauma, thereby improving postoperative recovery. Although BPS is considered the ideal choice for enhancing OLIF stability, it inevitably increases the operative time and intraoperative blood loss, thus raising the anesthesia risk for elderly patients. Additionally, posterior screw placement inevitably damages the paraspinal muscles and soft tissues, which may increase postoperative back pain, prolong bed rest, and affect recovery in elderly patients. Zhang et al. reported that compared with BPS, AF significantly reduced the operative time, intraoperative blood loss, and economic burden [25]. Liu et al. noted that AF maximally reduces the operative time, blood loss, radiation exposure, and soft tissue damage, achieving single‐stage interbody fusion and fixation through a small incision [8]. In this study, the AF + CA group showed significantly better results in terms of operative time, intraoperative blood loss, and postoperative VAS and ODI scores compared with the BPS group. Consequently, for elderly patients with lumbar degenerative diseases and conditions such as osteoporosis or osteopenia, AF + CA reduces surgical trauma, accelerates postoperative recovery, lowers anesthesia and surgery‐related complications, and provides clinicians with a safer and more efficient option when developing individualized treatment plans.

### 4.3 Cement Augmentation Did Not Improve the Interbody Fusion Rate

In this study, it was found that cement augmentation did not improve the interbody fusion rate. Previous studies have reported the application of cement‐augmented pedicle screws for treating degenerative lumbar diseases in elderly individuals undergoing TLIF. Song et al. reported that compared with conventional pedicle screws, cement‐augmented pedicle screws are more effective at treating osteoporotic thoracolumbar degenerative diseases, improving fusion rates and intervertebral height, and reducing screw loosening rates, thereby enhancing long‐term efficacy [11]. Wang et al. also suggested that cement‐augmented pedicle screws have greater fusion rates and lower screw loosening rates than conventional pedicle screws [12]. In the present study, no statistically significant difference in the 1‐year postoperative interbody fusion rate was observed among the three groups. Therefore, it is hypothesized that the effect of cement augmentation is more evident in reducing micromotion and subsidence in the short term, rather than in promoting long‐term bone fusion.

### 4.4 Risk Factors for Cage Subsidence Following OLIF Surgery

In this study, lower BMD was identified as a key risk factor for cage subsidence following OLIF. Previous studies have identified age, osteoporosis, endplate compromise, severe multifidus muscle degeneration, and higher intervertebral disc height as significant risk factors for cage subsidence following OLIF surgery [10, 27, 28, 29, 30]. In particular, osteoporosis has been recognized as one of the most important risk factors for cage subsidence. For example, Wang et al. noted that an increase in osteoporosis significantly increases the maximum stress on the endplates, thereby increasing the likelihood of cage subsidence [9]. Kotheeranurak et al. reported that having a BMD lower than −2.5 is a high‐risk factor for subsidence after OLIF [10]. Therefore, for osteoporotic patients undergoing OLIF surgery, clinicians should consider appropriate supplemental fixation or cement augmentation to reduce the risk of cage subsidence. Additionally, active postoperative anti‐osteoporotic treatment is crucial. For osteoporotic patients, the use of anti‐osteoporotic medications, including bisphosphonates, denosumab, or teriparatide, may be considered to enhance bone strength and reduce the likelihood of subsidence.

Diabetes was also identified as a significant contributor to cage subsidence in this analysis. In a previous study, diabetes was considered a factor, but the results did not indicate that it was a high‐risk factor for cage subsidence [28]. However, several potential mechanisms by which diabetes may predispose patients to subsidence have been hypothesized. Patients with diabetes, particularly those with poorly controlled long‐term glucose levels, often present with concurrent osteoporosis and reduced BMD, which decreases the ability of the bone to bear loads, thereby increasing the risk of cage subsidence [31]. Additionally, diabetes is associated with impaired bone healing, delayed osteogenesis, and hindered bone repair. Hyperglycemia is known to suppress osteoblastic activity and reduce bone formation, making it more difficult to establish a solid osseous bridge between the cage and the vertebral body post‐implantation, further raising the risk of subsidence [32]. Moreover, microvascular complications commonly observed in diabetic patients impair tissue perfusion, which slows bone healing and may compromise the stability of the fusion construct [33]. Chronic low‐grade inflammation, which is frequently present in patients with diabetes, can also disrupt the bone–cage interface, weakening the long‐term stability of the implant [34]. Given these concerns, strict perioperative glucose control is essential in diabetic patients to minimize the risk of complications and reduce the likelihood of cage subsidence.

### 4.5 Limitations and Prospect

In this study, potential confounders such as age, gender, and BMD were included as covariates in the multivariate model to minimize their impact on the primary outcomes and enhance the reliability of the findings. Future research should consider matching participants based on these key factors to further reduce their influence and improve outcome precision. Subgroup analyses were conducted to evaluate the effectiveness of cement augmentation across different BMD levels, as well as in patients with diabetes and various types of degenerative lumbar diseases (Tables S1 and S2). In individuals with osteopenia, the AF group showed a significantly higher rate of cage subsidence compared with the BPS group, while no significant differences were observed among groups in patients with osteoporosis. No notable differences in subsidence rates were found between diabetic and non‐diabetic patients across the three groups. For patients with spinal stenosis, the AF group had a significantly higher subsidence rate than both the AF + CA and BPS groups, with no significant difference between the latter two groups. No significant differences were detected among the groups for lumbar disc herniation or spondylolisthesis. However, given the study's limited sample size, detecting subtle differences among the groups may have been challenging. Future prospective studies with larger cohorts, particularly focusing on populations with osteoporosis and specific conditions such as spinal stenosis, are warranted to validate the role of cement augmentation in OLIF with AF.

This study has several limitations that need to be acknowledged. First, the retrospective nature and moderate sample size of the study may limit the generalizability of the findings and may not be sufficient to detect subtle differences between the groups. Second, the follow‐up period of 1 year, although adequate for initial assessment, might not be long enough to capture long‐term outcomes and complications such as delayed subsidence or fusion failure. Additionally, potential confounding factors, such as age, gender, BMD, variations in surgical techniques, and patient compliance with postoperative care, could have influenced the outcomes. While our multivariate analysis adjusted for several confounding variables, we recognize that a retrospective design inherently limits the ability to fully control for all potential confounders. Future studies with larger sample sizes, longer follow‐up periods, and more rigorous control of confounding factors are needed to confirm these findings and to provide a more comprehensive evaluation of the effectiveness of cement augmentation in OLIF combined with AF for treating degenerative lumbar diseases in elderly individuals.

## 5 Conclusion

AF + CA is effective in preventing cage subsidence in OLIF surgeries, offering benefits similar to BPS, with advantages such as a shorter surgical time, reduced intraoperative blood loss, and improved early postoperative outcomes. These findings support AF + CA as a viable alternative to BPS, particularly beneficial for elderly patients with degenerative lumbar diseases. AF + CA is recommended for elderly patients with comorbidities who may not tolerate longer surgical times or greater blood loss associated with BPS.

## Author Contributions

Conceptualization: Cong Jin and Weiqi Han. Methodology: Cong Jin and Lei He. Investigation: Cong Jin, Lei He, Fei Wang, and Xiaofeng Zhao. Formal analysis: Cong Jin and Weiqi Han. Writing – original draft: Cong Jin and Weiqi Han. Writing – review and editing: Lei He and Fei Wang. All authors had full access to the data in the study and took responsibility for the integrity of the data and the accuracy of the data analysis.

## Ethics Statement

The authors confirmed that the study was performed in accordance with the Declaration of Helsinki. The study was approved by the Ethics Committee (full name: Ethics Committee of the Shaoxing University Affiliated First Hospital, reference number No. 202405201), and informed consent was obtained from all patients.

## Conflicts of Interest

The authors declare no conflicts of interest.

## Supporting information


**Table S1.** Subgroup analysis of cage subsidence rates in three groups based on bone mineral density and diabetes history.

**Table S2.** Subgroup analysis of cage subsidence rates in three groups based on different types of degenerative lumbar diseases.
