# Supplementary material for: Oblique Lumbar Interbody Fusion Combined With Anterolateral Fixation and Cement Augmentation for the Treatment of Degenerative Lumbar Diseases in the Elderly Population: A Retrospective Study
Source: Orthop Surg. 2024 Dec 3;17(2):446–59. doi: 10.1111/os.14315 (PMC11787981; doi:10.1111/os.14315)
Supplement: Supplementary file 1 — Table S1. Subgroup analysis of cage subsidence rates in three groups based on bone mineral density and diabetes history. [file OS-17-446-s002.docx]

**Supplementary Table 1: Subgroup analysis of cage subsidence rates in three groups based on bone mineral density and diabetes history.**

a: P< 0.05, AF group compared with the BPS group.

AF+CA: Anterolateral fixation and cement augmentation; AF: Anterolateral fixation; BPS: Bilateral pedicle screw fixation; CS: Cage subsidence; Non-CS: Non-cage subsidence.

| **Characteristics** | **AF+CA** | **AF** | **BPS** |
| --- | --- | --- | --- |
| **Osteopenia** |  |  |  |
| n | 27 | 31 | 82 |
| Cage Subsidence, n (%) |  |  |  |
| CS | 6 (4.3%) | 14 (10%) ^a^ | 20 (14.3%) |
| Non-CS | 21 (15%) | 17 (12.1%) | 62 (44.3%) |
| **Osteoporosis** |  |  |  |
| n | 10 | 10 | 20 |
| Cage Subsidence, n (%) |  |  |  |
| CS | 3 (7.5%) | 6 (15%) | 11 (27.5%) |
| Non-CS | 7 (17.5%) | 4 (10%) | 9 (22.5%) |
| **Diabetic** |  |  |  |
| n | 6 | 7 | 18 |
| Cage Subsidence, n (%) |  |  |  |
| CS | 2 (6.5%) | 5 (16.1%) | 7 (22.6%) |
| Non-CS | 4 (12.9%) | 2 (6.5%) | 11 (35.5%) |
| **Non-diabetic** |  |  |  |
| n | 31 | 34 | 84 |
| Cage Subsidence, n (%) |  |  |  |
| CS | 7 (4.7%) | 15 (10.1%) | 24 (16.1%) |
| Non-CS | 24 (16.1%) | 19 (12.8%) | 60 (40.3%) |
