# Supplementary material for: Oblique Lumbar Interbody Fusion Combined With Anterolateral Fixation and Cement Augmentation for the Treatment of Degenerative Lumbar Diseases in the Elderly Population: A Retrospective Study
Source: Orthop Surg. 2024 Dec 3;17(2):446–59. doi: 10.1111/os.14315 (PMC11787981; doi:10.1111/os.14315)
Supplement: Supplementary file 2 — Table S2. Subgroup analysis of cage subsidence rates in three groups based on different types of degenerative lumbar diseases. [file OS-17-446-s001.docx]

**Supplementary Table 2: Subgroup analysis of cage subsidence rates in three groups based on different types of degenerative lumbar diseases.**

a: P< 0.05, AF+CA group compared with the AF group. b: P< 0.05, AF group compared with the BPS group. AF+CA: Anterolateral fixation and cement augmentation; AF: Anterolateral fixation; BPS: Bilateral pedicle screw fixation; CS: Cage subsidence; Non-CS: Non-cage subsidence.

| **Characteristics** | **AF+CA** | **AF** | **BPS** |
| --- | --- | --- | --- |
| **Spinal stenosis** |  |  |  |
| n | 23 | 24 | 64 |
| Cage Subsidence, n (%) |  |  |  |
| CS | 5 (4.5%) ^a^ | 14 (12.6%) ^b^ | 20 (18%) |
| Non-CS | 18 (16.2%) | 10 (9%) | 44 (39.6%) |
| **Lumbar disc herniation** |  |  |  |
| n | 5 | 7 | 9 |
| Cage Subsidence, n (%) |  |  |  |
| CS | 2 (9.5%) | 1 (4.8%) | 2 (9.5%) |
| Non-CS | 3 (14.3%) | 6 (28.6%) | 7 (33.3%) |
| **Spondylolisthesis** |  |  |  |
| n | 9 | 10 | 29 |
| Cage Subsidence, n (%) |  |  |  |
| CS | 2 (4.2%) | 5 (10.4%) | 9 (18.8%) |
| Non-CS | 7 (14.6%) | 5 (10.4%) | 20 (41.7%) |
